# Monkeypox Virus in Wastewater Samples from Santiago Metropolitan Region, Chile

**DOI:** 10.3201/eid2911.230096

**Published:** 2023-11

**Authors:** Manuel Ampuero, Constanza Martínez-Valdebenito, Marcela Ferrés, Ricardo Soto-Rifo, Aldo Gaggero

**Affiliations:** Universidad de Chile, Santiago, Chile (M. Ampuero, R. Soto-Rifo, A. Gaggero);; Pontificia Universidad Católica de Chile, Santiago (C. Martínez-Valdebenito, M. Ferrés);; Millennium Institute on Immunology and Immunotherapy, Santiago (R. Soto-Rifo)

**Keywords:** monkeypox virus, MPXV, viruses, mpox, wastewater samples, sewage, infectivity, wastewater-based epidemiology, zoonoses, metropolitan region, Santiago, Chile

## Abstract

Sewage surveillance provides useful epidemiologic and public health information on viral infections at the population level. We detected monkeypox virus DNA from sewage samples covering 85% of the population in Santiago Metropolitan Region Chile. We also isolated infective viruses from those samples. Wastewater surveillance could complement clinical surveillance for monkeypox virus.

On May 13, 2022, the World Health Organization raised an alert caused by a large increase in the number of infections by monkeypox virus (MPXV), which causes mpox, a zoonotic disease endemic to some countries of Central and West Africa that has rapidly expanded to nonendemic countries (*1*). A case of MPXV infection in Chile was confirmed on July 17, and since then, >1,400 cases and 2 deaths related to mpox have been reported during the outbreak, according to the Chile Ministry of Health (*2*). The Santiago Metropolitan Region in Chile is the most populated region in the country, accounting for >40% of the total population and most (81%) of the reported MPXV infections (*2*).

Wastewater surveillance has been demonstrated as a key contributor in monitoring viruses, such as poliovirus and SARS-CoV-2, enabling tracking of new variants and, thus, providing an accurate view of infections at the population level (*3*–*5*). In this regard, MPXV detection in sewage samples has also been proposed as a useful complement to clinical surveillance (*6*–*9*). Because stigma and discrimination associated with certain infections limit the willingness of at-risk persons to consult hospital centers, wastewater-based epidemiology (WBE) becomes even more useful because anonymous pooled samples enable visualization of the contributions of a community without revealing individual identities (*10*).

We report wastewater monitoring of MPXV DNA in sewage samples from 3 wastewater treatment plants (WWTPs), accounting for 85% of the overall sewage from Santiago Metropolitan Region, representative of >5.5 million persons. We also report the presence of infective MPXV in those samples.

## The Study

We collected 21 raw samples of wastewater during April‒September 2022 from the WWTPs El Trebal (n = 6), La Farfana (n = 6), and La Higuera (n = 9). We collected samples in 1,000-mL sterile propylene flasks, transported them to the Laboratory of Environmental Virology at Universidad de Chile Faculty of Medicine, and stored them at 4°C until processing.

We concentrated the samples by ultracentrifugation according to the protocol described by Fumian et al. (*11*). We resuspended the pellet obtained in 200 μL of phosphate-buffered saline and stored at −80°C until use.

We used 200 µL of concentrated viral particles to isolate DNA with the QIAamp DNA MiniKit (QIAGEN, https://www.qiagen.com), according to the instructions provided by the supplier. We mixed 5 µL of DNA with the TaqMan Microbe Detection MonkeyPox Vi07922155_s1 (ThermoFisher Scientific, https://www.thermofisher.com) and the TaqPath 1-Step Multiplex Master Mix (ThermoFisher Scientific) for the specific detection of MPXV DNA in a QuantStudio 5 real-time PCR machine (ThermoFisher Scientific). A pMG-Amp plasmid carrying the synthetic MPXV DNA fragment 5′-GTGTCTGAATCGTTCGATTAACCCAACTCATCCATTTTCAGATGAATAGAGTTATCGATTCAGACACATGCTTTGAGTTTTGTTGAATCGATGAGTGAAGTATCATCGGTTGCACCTTCAGATGC-3′, which contains the target sequence of the primers, was synthesized at Macrogen Inc. (https://www.macrogen.com). We used that plasmid as a positive control and as a template for the calibration curve enabling the quantification of MPXV genome copies per milliliter. We cloned the amplified DNA fragment into pGEM-T Easy Vector (Promega, https://www.promega.com) and transformed it into *Escherichia coli* JM109. Five clones from each sample were sequenced at Macrogen Inc. and compared with MPXV sequences from the 2022 outbreak.

From the 21 sewage samples collected and analyzed from the 3 WWTPs, we detected MPXV DNA in 6 (Table). Consistent with earlier cases of mpox reported in Chile, viral DNA was detected in sewage samples collected in July (La Farfana), August (La Farfana, El Trebal) and September (La Farfana, El Trebal, La Higuera), but not in April or May (Table).

**Table:** Monkeypox virus detection in wastewater samples from 3 areas, Santiago Metropolitan Region, Chile, 2022*

**Date **	La Farfana	El Treba**l**	La Higuera
**Apr 11**	ND	ND	‒
**Apr 19**	‒	‒	ND
**Apr 22**	ND	ND	‒
**May 5**	ND	ND	‒
**May 16**	‒	‒	ND
**May 19**	ND	ND	‒
**Jun 6**	‒	‒	ND
**Jun 16**	ND	ND	‒
**Jul 18**	36	‒	ND
**Jul 22**	ND	ND	‒
**Aug 12**	ND	ND	‒
**Aug 16**	35	171	ND
**Aug 18**	ND	ND	‒
**Sep 15**	ND	ND	895
**Sep 20**	2,231	960	ND

*Values are genome copies/mL. ND, not done; ‒, negative.

**Figure 1:**
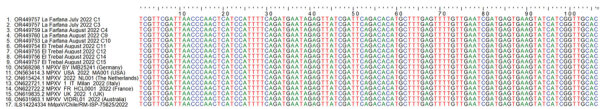
Comparison of nucleotide sequences of the MPXV amplicon obtained for wastewater samples from Santiago Metropolitan Region, Chile (sewage samples 1‒9), with reference sequences obtained from other countries during the 2022 mpox outbreak. The 106-bp amplicon generated by quantitative reverse transcription has 100% homology with MPXV sequences obtained in 2022 from cases reported by different countries. GenBank numbers and location and date of isolation are provided for the 9 Chile sample sequences obtained in this study; GenBank or GISAID (https://www.gisaid.org) accession numbers and country are provided for reference sequences. MPXV, monkeypox virus.

Quantification of MPXV DNA in sewage showed viral loads ranging from 35 to 2,231 genome copies/mL (Table). Higher viral loads in sewage samples correlated with an increase in the number of cases reported by the Chile Ministry of Health in Santiago. Sequencing of the 106-bp amplified DNA fragment from wastewater samples showed 100% homology with MPXV sequences from the 2022 outbreak reported from Germany, the Netherlands, Italy, France, the United Kingdom, the United States, and Chile (Figure 1).

To determine whether the sewage samples contained viable MPXV, we used the samples that had the highest viral load to inoculate VeroE6 monolayers (ATCC CRL-1586). For this procedure, we infected cells with a mixture of MPXV DNA–positive sewage samples and culture medium and collected the supernatant after 7 days for PCR detection. We stored the remaining supernatant and performed a second round of infection by using the supernatant from the first infection. We used positive and negative controls in separate plates to avoid cross-contamination. At 24- and 48-hours postinfection, we collected supernatant for MPXVDNA detection by PCR.

In addition, we inoculated 300 µL of sample AF0922 supplemented with 700 µL of Dulbecco’s Modified Eagle Medium plus 2% fetal bovine serum (FBS) into VeroE6 cells in a 6-cm plate. After 2 hours of incubation, we replaced the medium with 5 mL of Dulbecco’s Modified Eagle Medium plus 2% FBS. After 7 days, we fixed infected cells with 4% glutaraldehyde and performed negative staining for electron microscopy observation (Appendix Figure) All procedures related to viral isolation were performed in a Biosafety Level 3 laboratory at Unidad de Virología Aplicada, Pontificia Universidad Católica de Chile, Santiago.

**Figure 2:**
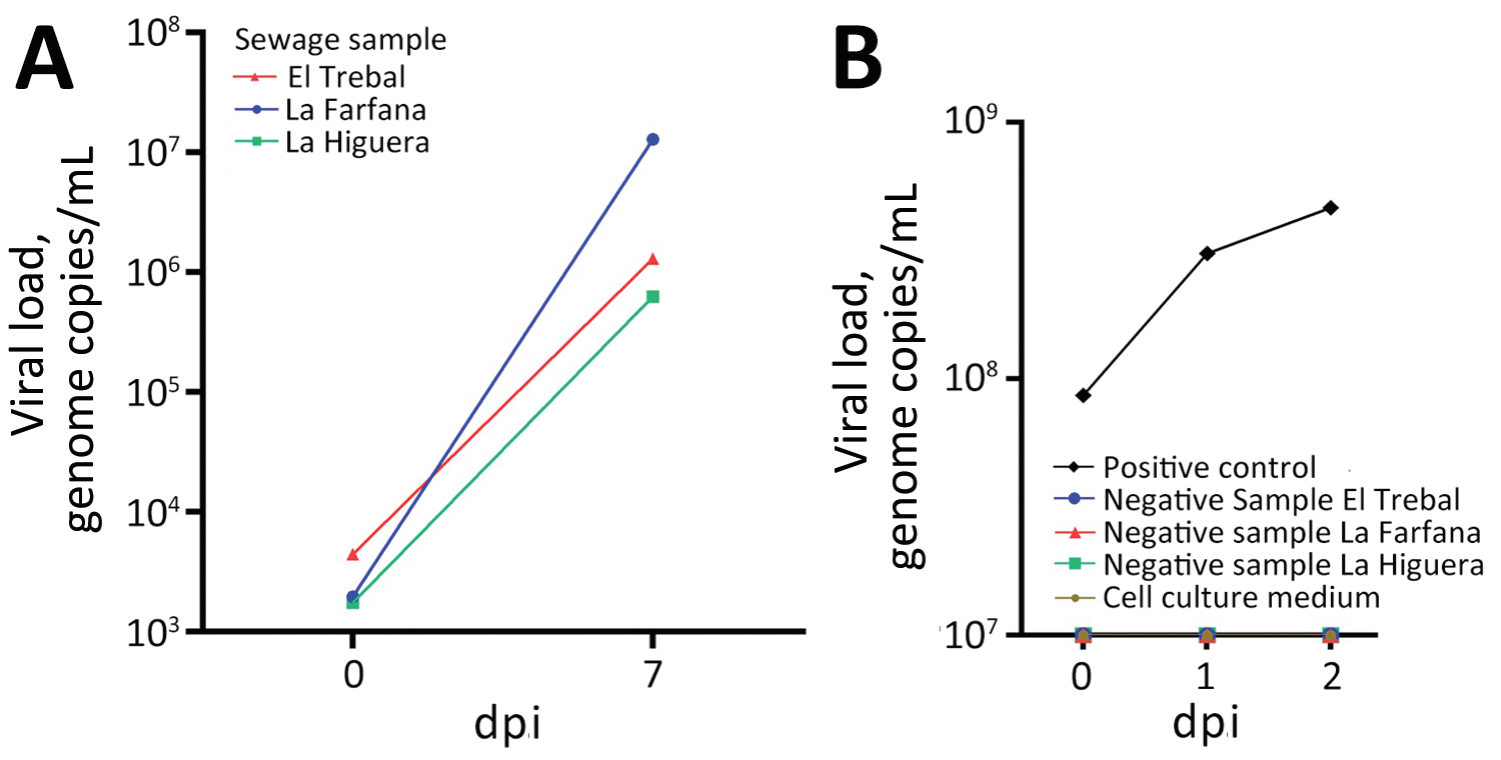
Detection of viral genome of monkeypox virus in wastewater samples from wastewater treatment plants in Santiago Metropolitan Region, Chile. A) PCR results for Vero E6 cell supernatant at 7 dpi. B) PCR results for supernatant samples of Vero E6 cells infected with positive control (cell culture supernatant infected with monkeypox virus) and negative controls (Dulbecco’s Modified Eagle Medium plus 2% fetal bovine serum and negative wastewater samples). dpi, days postinfection.

We detected a high viral DNA load in the supernatant at day 7 postinoculation, suggesting the presence of infective MPXV in sewage samples (Figure 2, panel A). We were not able to detect MPXV DNA from cells inoculated with samples that tested negative for the virus (Figure 2, panel B). Electron microscopy analyses of VeroE6 cells inoculated with MPXV recovered from sample AF0922 showed intracellular viral particles with an average size of ≈300 nm (Appendix Figure).

## Conclusions

WBE has acquired an increasingly useful role in surveillance systems that efficiently detect of pathogenic microorganisms. It will also be useful as a tool for control and timely prevention of endemic and emerging infectious diseases.

Using WBE as a complement to universal clinical surveillance enables determination of actual pathogen circulation and its load in a population. For example, WBE has become a useful tool worldwide for visualizing the circulation of SARS-CoV-2 and its variants (*3*–*5*). Therefore, WBE could also complement clinical surveillance of MPXV, enabling estimation of actual circulation and load of the virus in a community (*6*,*9*). However, it will be useful to generate more information regarding virus elimination in an infected person; viral DNA load in stool, urine, semen, saliva, and other secretions; and persistence and infectivity of the virus in the environment and, in particular, in a matrix as complex as wastewater.

In conclusion, we detected MPXV DNA and determined its concentration in wastewater in Santiago, Chile. We were also able to isolate the virus from samples with the highest viral loads. Although detection of viable virus in sewage samples observed in this study generates an alert, there is no information on the risk that this could have for the personnel working in treatment plants. The potential risk for environmental transmission of MPXV is still unknown and thus remains a serious public health issue.

Appendix Additional information on monkeypox virus in wastewater samples from Santiago Metropolitan Region, Chile.
