# Supplementary material for: Monkeypox Virus in Wastewater Samples from Santiago Metropolitan Region, Chile
Source: Emerg Infect Dis. 2023 Nov;29(11):2358–61. doi: 10.3201/eid2911.230096 (PMC10617339; doi:10.3201/eid2911.230096)
Supplement: Appendix — Additional information on monkeypox virus in wastewater samples from Santiago Metropolitan Region, Chile. [file 23-0096-Techapp-s1.pdf]

Article DOI: https://doi.org/10.3201/eid2911.230096

EID cannot ensure accessibility for Supplemental Materials supplied by authors. Readers who have difficulty accessing supplementary content should contact the authors for assistance.

Monkeypox Virus in Wastewater Samples from Santiago Metropolitan Region, Chile

Appendix

Methods

Wastewater Treatment Procedure

Samples were concentrated by ultracentrifugation according to the protocol described by Fumian et al. (*1*). Briefly, 42 mL of each sample previously homogenized were ultracentrifuged at 100,000 × *g* for 1 hour at 4°C. The supernatant was discarded, and the pellet was resuspended in 3.5 mL of 0.25N glycine buffer, pH 9.5, on ice for 30 min. The solution was neutralized with 3.5 mL of 2× PBS, pH 7.2, and clarified by centrifugation at 12,000 × *g* for 15 min at 4°C. The supernatant enriched in viral particles was further concentrated by ultracentrifugation at 100,000 × *g*, and the pellet was resuspended in 200 µL of 1× PBS and stored at −80°C until use.

Infectivity Assay

To evaluate whether sewage samples contained viable monkeypox virus, we selected the samples containing the higher viral load from each WWTP and used them to inoculate VeroE6 (ATCC CRL-1586) monolayers. These cells were seeded at 90% confluence in a 6-well plate and inoculated the following day with a mix of 200 µL of monkeypox DNA-positive sewage samples and 200 µL of culture medium (DMEM, 2% FBS, 0.1 mg of penicillin/streptomycin, and 0.2 mg of neomycin). The mixture and VeroE6 cells were incubated for 1.5 hours, and then the mixture was replaced with 500 µL of fresh medium. Seven days later, we scraped and centrifuged the cells, discarded the pellet, and used 250 µL of the supernatant for PCR detection as described above. We stored the remaining supernatant. To avoid cross-contamination, we included positive and negative controls (DMEM, 2% FBS) in separate plates.

For a second round of infection, we seeded VeroE6 cells in a 12-well plate and added 100 µL of supernatants from the previous infection, along with 100 µL of DMEM, 2% FBS in triplicate. We included positive and negative controls (DMEM, 2% FBS and negative sewage samples) in a separate plate. At 24h and 48h postinfection, we collected 250 µL of supernatant for monkeypox virus DNA detection by PCR.

References

1. Fumian TM, Leite JP, Castello AA, Gaggero A, Caillou MS, Miagostovich MP. Detection of rotavirus A in sewage samples using multiplex qPCR and an evaluation of the ultracentrifugation and adsorption-elution methods for virus concentration. J Virol Methods. 2010;170:42–6. PubMed https://doi.org/10.1016/j.jviromet.2010.08.017

2. Reynolds ES. The use of lead citrate at high pH as an electron-opaque stain in electron microscopy. J Cell Biol. 1963;17:208–12. PubMed https://doi.org/10.1083/jcb.17.1.208

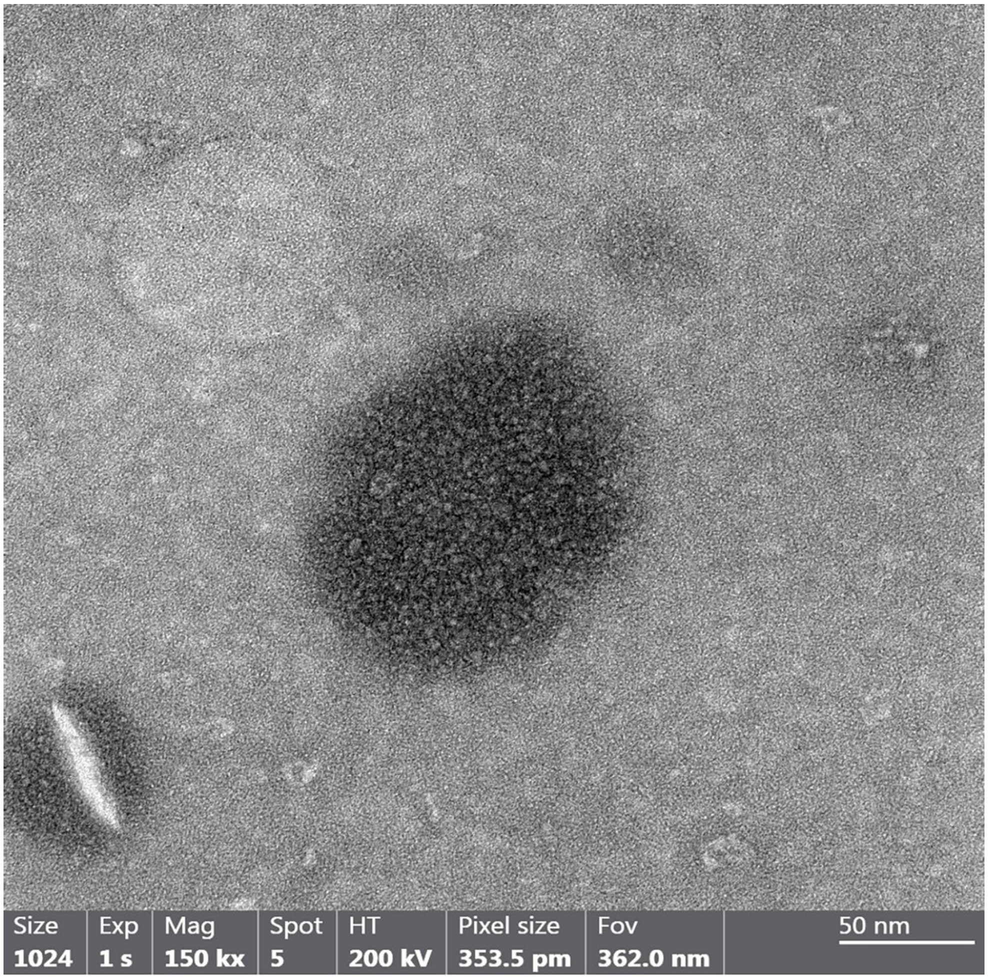

Appendix Figure. Transmission electron microscopy of cultured monkeypox virus from MPXV DNA-positive sewage samples. VeroE6 cells were inoculated with MPXV DNA-positive sample for 7 days, after cytopathic effect was visible. The monolayer was fixed for 18 h in 2.5% glutaraldehyde, 0.1M sodium cacodylate buffer, pH 7.2, postfixed in 1% osmium tetroxide in cacodylate buffer, and washed with distilled water. Samples were stained in block with 2% aqueous uranyl acetate, dehydrated with a series of acetone, and pre-filtered in 1:1 resin:acetone. Samples were placed in pure resin for 4 h (at room temperature) and then embedded in molds with pure resin and polymerized (60°C for 48 h). Thin sections were obtained on a Leica Ultracut R ultramicrotome, placed on copper grids, and stained with 2% aqueous uranyl acetate for 8 minutes and with lead citrate according to Reynolds (*2*) for 4 minutes. The sections were observed in a Thermo-Fisher Talos F200C transmission electron microscope at 200kV (Advanced Microscopy Unit, FCB, Catholic University of Chile). Scale bar indicates 50 nm.
